# Choroidal Mast Cells and Their Degranulation Are a Pivotal Trigger for Myopia Development

**DOI:** 10.1167/iovs.66.14.22

**Published:** 2025-11-12

**Authors:** Jue Shi, Shin-ichi Ikeda, Tomokazu Fukuchi, Junhan Chen, Kate Gettinger, Satoshi Imanishi, Kazuno Negishi, Kazuo Tsubota, Toshihide Kurihara

**Affiliations:** 1 Laboratory of Photobiology, Department of Ophthalmology, Keio University School of Medicine, Shinjuku-ku, Tokyo, Japan; 2 Department of Ophthalmology, Keio University School of Medicine, Shinjuku-ku, Tokyo, Japan; 3 Tsubota Laboratory, Inc., Shinjuku-ku, Tokyo, Japan

**Keywords:** myopia, choroid, mast cell, lens-induced myopia

## Abstract

**Purpose:**

Mast cells (MCs), key immune regulators, contribute to inflammatory responses through their degranulation. Choroidal thinning is a hallmark of myopia, and MC degranulation has been shown to induce choroidal thinning in a rat model of geographical atrophy. However, the role of choroidal MCs in myopia remains poorly understood. This study aimed to demonstrate that an increased number and proportion of degranulated MCs in the choroid are important determinants of choroidal thinning and myopia development.

**Methods:**

Lens-induced myopia (LIM) was induced by monocular −30 diopter (D) lens wearing in C57BL/6 mice, MC-deficient mice (*Mcpt5*/*Cma1^DTR^*^/+^), and MC-deficient mice receiving suprachoroidal injection of peritoneal MCs (PMCs). Cromolyn sodium (cromolyn) and pemirolast potassium (pemirolast), reagents that inhibit degranulation by stabilizing MC membranes, were topically applied in LIM mice. Ocular parameters including refraction, axial length, and choroidal thickness were measured before and after LIM. Choroidal MC degranulation was evaluated by choroidal flatmount staining.

**Results:**

LIM was successfully induced in wild-type mice but was significantly suppressed in MC-deficient mice. Suprachoroidal injection of PMCs into MC-deficient mice restored myopia progression, as evidenced by axial elongation and choroidal thinning. MC degranulation was significantly increased by −30 D lens wearing, whereas topical administration of cromolyn and pemirolast effectively inhibited both MC degranulation and myopia progression.

**Conclusions:**

Choroidal MC degranulation plays a critical role in LIM by promoting choroidal thinning and axial elongation. These findings enhance our understanding of the role of choroidal MC degranulation in myopia, suggesting a potential therapeutic target for myopia treatment.

Myopia, also known as nearsightedness, is a common refractive error that has evolved into a serious public health concern worldwide, affecting an increasing proportion of the population.^1^^,^^2^ The primary cause of myopia is axial elongation, where the length of the eyeball no longer corresponds with the focusing power of the lens and cornea, causing light from distant objects to be focused in front of the retina, thereby resulting in blurred vision.^3^ A recent study has indicated that genetics, environment, and lifestyle are major risk factors for myopia.^4^ Although several effective interventions for myopia control have been introduced in recent years, the molecular mechanisms underlying its progression remain poorly understood.^5^^,^^6^

The choroid, a highly vascularized layer of the eye located between the retinal pigment epithelium (RPE) and sclera, supplies oxygen and nutrients to the outer retina and supports immune surveillance via resident immune cells, including dendritic cells, macrophages, and mast cells (MCs).^7^ Choroidal thickness (ChT) has emerged as a critical biomarker and potential driver of myopia progression.^8^^–^^10^ Experimental studies in mice, guinea pigs, and chicks have shown that myopic defocus induces choroidal thickening and inhibits ocular elongation, whereas hyperopic defocus causes choroidal thinning and promotes elongation.^11^^–^^13^ Clinical observations have also demonstrated a similar inverse correlation between ChT and axial length (AL) in high myopia.^14^ Recently, we demonstrated that RPE-specific suppression of vascular endothelial growth factor (VEGF) induced choroidal thinning and led to axial elongation, whereas VEGF overexpression resulted in choroidal thickening and reduced AL, indicating that ChT directly regulates AL.^8^

MCs are tissue-resident immune cells primarily localized near blood vessels, nerves, and tissue interfaces such as skin and gastrointestinal tract.^15^ They are characterized by multiple cytoplasmic granules, including histamine, proteases, and cytokines.^16^ MCs are crucial in immune responses and tissue homeostasis through degranulation, a process that rapidly releases these mediators into the extracellular space.^17^^,^^18^ Increasing evidence implicates MCs in a wide range of conditions, including allergic diseases, autoimmune dysfunctions, and cancer.^19^ In the eye, MCs are particularly abundant in the conjunctiva, where they play a pivotal role in the pathogenesis of allergic conjunctivitis (AC).^20^ Moreover, MCs are widely distributed in the choroid across multiple species, including humans, mice, rats, birds, and fish.^21^ Previous studies have indicated that increased MCs and degranulation are associated with choroidal thinning in a rat model with geographic atrophy (GA).^22^^,^^23^ Given that choroidal thinning is a hallmark of myopia, we hypothesized that choroidal MCs contribute to myopia development by promoting choroidal thinning through degranulation.

In this study, we demonstrated that elevated quantities and proportions of degranulated MCs in the myopic choroid serve as key determinants of both choroidal thinning and myopia progression, utilizing a lens-induced myopia (LIM) model combined with MC-deficient and MC-reconstituted murine models.

## Methods

### Animals

All animal experiments were approved by the Animal Experimental Committee of Keio University (A2022-242) and adhered to the Institutional Guidelines on Animal Experimentation at Keio University, the ARVO Statement for the Use of Animals in Ophthalmic and Vision Research, and the Animal Research: Reporting of In Vivo Experiments (ARRIVE) guidelines for the use of animals in research.

The genetic background of all mice in our study was C57BL/6. The wild-type (WT) and *Mcpt5*/*Cma1^DTR^*^/+^ mice were purchased from the Central Laboratory for Experimental Animals Japan (Tokyo, Japan) and RIKEN Bioresource Research Center (Tsukuba, Japan), respectively.^24^ The mice were housed in transparent cages in a 12-hour light/dark cycle room at a temperature of 22°C ± 3°C. The population for each cage was limited to five mice or fewer.

Postnatal day 18 (P18), *Mcpt5*/*Cma1^DTR^*^/+^ mice were administered diphtheria toxin (DT; 250 ng/mouse/d IP, #048-34371; Fujifilm Wako, Osaka, Japan) for 7 consecutive days to generate MC-deficient mice.^24^ WT littermates receiving the same DT treatment served as controls.

### LIM Mouse Model

According to a previously reported protocol, a frame designed with a −30 diopter (D) lens for mice was placed in front of the right eye, and a frame without a lens was placed in front of the left eye as an internal control.^25^ The lenses were cleaned three times per week. Body weight (BW) was measured before and after the 3-week lens-wearing period.

### Ocular Parameters Measurements

Ocular parameters were measured as previously described.^8^^,^^26^ Pupil dilation and ciliary muscle paralysis were achieved by applying Medrolin eye drops (Santen Pharmaceutical Co., Osaka, Japan) to the mouse eyes to ensure accurate measurements. Following successful dilation, a combination of midazolam (4 mg/kg; Sandoz K.K., Tokyo, Japan), medetomidine (0.75 mg/kg; Orion Corporation, Espoo, Finland), and butorphanol tartrate (5 mg/kg; Meiji Seika Pharma Co., Ltd., Tokyo, Japan), referred to as MMB solution, was used to anesthetize the mice. Refraction was measured by an infrared photorefractor (Steinbeis Transfer Center, Stuttgart, Germany), and the corneal radius of curvature (CRC) was measured by another infrared photorefractor (Steinbeis Transfer Center). The central corneal thickness (CCT), anterior chamber depth (ACD), lens thickness (LT), vitreous chamber depth (VCD), AL, ChT, and retinal thickness (RT) were measured using spectral-domain optical coherence tomography (SD-OCT; Envisu R4310 imaging system; Leica Microsystems, Wetzlar, Germany). The AL was the distance between the corneal vertex and macular fovea of the RPE, and the ChT was defined as the area between the RPE boundary and the posterior surface of the choroid.^27^ The CCT was defined as the distance from the central anterior corneal surface to the central posterior corneal surface, the ACD was the distance from the posterior corneal surface to the anterior lens surface, the LT was defined as the distance between the anterior and posterior poles of the lens along the optical axis, the VCD was the distance between the posterior surface of the lens and the RPE boundary, and the RT was the area between the inner and outer boundaries of the retina.^28^ ChT and RT measurements were acquired using ImageJ 1.52v11 (National Institutes of Health, Bethesda, MD, USA).^8^

### Electroretinography Analyses

The dark-adapted electroretinography was recorded as previously described.^29^ After dark adaption for 12 hours, mice were anesthetized with MMB under dim red light and dilated with Medrolin. Contact lens electrodes (Mayo Corporation, Inazawa, Japan) were placed on each cornea, with a subcutaneous reference and a tail-clipped electrode serving as the ground. Utilizing a Ganzfeld dome, an acquisition system (PuREC; Mayo Corporation), and light-emitting diode (LED) stimulus, scotopic responses from each eye were elicited by full-field light stimuli (LS-100; Mayo Corporation) at intensities of 0.02, 0.1, 0.5, 2, and 10 log cd·s/m^2^. The low pass of the amplifiers was set to 30 Hz. Amplitudes of a-waves and b-waves were analyzed.

### Histochemistry

Tissues, including ear skin, abdominal skin, and tongue, were isolated after euthanasia, fixed overnight at 4°C in 4% paraformaldehyde (PFA; #168-23255; Fujifilm Wako) on a rocker, embedded in paraffin, and sectioned at 5 µm using a microtome (REM-710; Yamato Kohki, Saitama, Japan). Subsequently, sections were deparaffinized, rehydrated with distilled water, and stained with 0.1% toluidine blue solution (#36693.02; Serva Electrophoresis GmbH, Heidelberg, Germany) to detect MCs. Samples were then washed, dehydrated and visualized under a microscope (BX53; Olympus, Tokyo, Japan).

### Immunofluorescence

The mice were euthanized, and their eyes were enucleated for choroidal flatmount immunostaining. The eyeballs were cleaned of muscle, fat, and conjunctival tissues following washing in PBST: phosphate-buffered saline (PBS) containing 2% Triton X-100 (#12967; Nacalai Tesque, Inc., Kyoto, Japan). Subsequently, eyeballs were fixed in 4% PFA for 1 hour on a rocker at room temperature. Samples were bleached using a Melanin Bleach Kit (#24883; Polysciences, Warrington, PA, USA), after which the cornea, lens, and retina were removed to isolate choroidal tissues. PBST solution containing 5% bovine serum albumin (BSA; #3013-15104; Fujifilm Wako) served as the blocking buffer. The choroidal flatmounts were incubated overnight at 4°C in the blocking buffer with or without the primary antibody anti-MC chymase antibody (1:500, #ab233103; Abcam, Cambridge, UK), followed by overnight incubation with Donkey anti-Rabbit Secondary Antibody (1:500, #A-31572; Thermo Fisher Scientific, Waltham, MA, USA) with light blocking. Finally, the choroidal flatmounts were washed and mounted. Choroidal MCs were visualized with a fluorescence microscope (BZ-X800; Keyence, Osaka, Japan), and high-resolution images were acquired using a confocal microscope (LSM 980; ZEISS, Oberkochen, Germany). The number of MCs was counted manually, and the degranulation ratio was calculated as the proportion of degranulated MCs divided by the total number of MCs in each flatmount.

### Mouse Peritoneal MC Isolation, Staining, and Administration

As previously described, peritoneal MCs (PMCs) were isolated from the peritoneum of 8-week-old male WT mice and cultured in RPMI 1640 medium (#61870036; Thermo Fisher Scientific) containing 20% fetal bovine serum (#10270106; Thermo Fisher Scientific) and 1% penicillin (#09367-34; Nacalai Tesque, Inc.), supplemented with recombinant murine IL-3 (10 ng/mL; #213-13; PeproTech, Cranbury, NJ, USA) and recombinant murine stem cell factor (SCF; 30 ng/mL, #250-03; PeproTech).^30^ After 3 weeks of incubation, PMCs were proliferated and their purity was determined by flow cytometry using APC anti-mouse CD117 (1:200, #17-1171-82; Thermo Fisher Scientific) and PE anti-mouse FcεRIα (1:200, #1134308; Biolegend, San Diego, CA, USA) on a flow cytometer (CytoFLEX S; Beckman Coulter, Brea, CA, USA).

PMCs were fixed with 4% PFA at room temperature on a rocker for in vitro staining. Cells were blocked in PBS containing 5% BSA and 0.2% Triton X-100, then incubated overnight at 4°C with anti-MC chymase antibody (1:500) in blocking buffer. After washing, cells were incubated for 1 hour at room temperature on a rocker with the corresponding secondary antibodies and 4′,6-diamidino-2-phenylindole (DAPI; 1:10,000, #PK-CA707-40043; PromoCell, Heidelberg, Germany) with light blocking. Finally, cells were washed and imaged using the fluorescence microscope.

PMCs were suspended in PBS at a density of 5 × 10^5^ cells per 100 µL, and 1 µL containing 5 × 10^3^ PMCs was injected into the suprachoroidal space (SCS) in both eyes in *Mcpt5*/*Cma1^DTR^*^/+^ mice using a microliter syringe. As controls, the same volume of PBS was injected into WT and *Mcpt5*/*Cma1^DTR^*^/+^ mice. Seven days after injection, mice were euthanized, and choroidal tissues were collected for PMC staining with the anti-MC chymase antibody, followed by examination using the fluorescence microscope.

### Drug Administration

From 3 to 6 weeks of age, both LIM and non-LIM WT mice received topical administration of 10 µL of 4% cromolyn sodium (cromolyn; #C0399; Sigma-Aldrich, St. Louis, MO, USA) and 0.1% pemirolast potassium (pemirolast; Santen Pharmaceutical Co.) eye drops to both eyes once daily between 2:00 PM and 4:00 PM. PBS was administered in the control group. In non-LIM mice, retinal function and ocular parameters including CRC, CCT, ACD, LT, VCD, and RT were measured; in LIM mice, refraction, AL, and ChT were evaluated before and after eye drop treatment.

### Western Blotting

Choroidal and scleral tissues were isolated after euthanizing the mice and were homogenized in radioimmunoprecipitation assay buffer (#89900; Thermo Fisher Scientific). Protein concentrations were measured using a bicinchoninic acid (BCA; #23210; Thermo Fisher Scientific) protein assay and adjusted using Laemmli sample buffer (#09499-14; Nacalai Tesque, Inc.). Samples, together with a protein-size marker (#LC5603; Thermo Fisher Scientific), were separated by sodium dodecyl sulfate–polyacrylamide gel electrophoresis (SDS-PAGE) on 12% acrylamide gels (#1610175; Bio-Rad Laboratories, Hercules, CA, USA). Proteins were transferred onto polyvinylidene fluoride (PVDF) membranes (#162-0177; Bio-Rad Laboratories) and blocked with Blocking One (#03953; Nacalai Tesque, Inc.). For choroidal lysates, membranes were incubated overnight at 4°C with Anti-MC chymase antibody (1:10,000), anti-MC mcpt6 (tryptase) antibody (1:10,000, #MAB3736; Bio-Techne, Minneapolis, MN, USA), and β-actin mAb (1:5000, #3700; Cell Signaling Technology, Danvers, MA, USA). For scleral lysates, membranes were incubated with anti-COL1A1 (1:5000, #72026T; Cell Signaling Technology), anti-MMP2 (1:5000, #4022S; Cell Signaling Technology), and β-actin mAb. After incubation with appropriate horseradish peroxidase–conjugated secondary antibodies (1:10,000), proteins were detected using SuperSignal West Femto Maximum Substrate (LAS-4000 mini; GE Healthcare, Chicago, IL, USA).

### Statistics

An unpaired two-tailed *t*-test, a one-way ANOVA with least significant difference (LSD) post hoc test, and one- and two-way ANOVA with Tukey's post hoc tests were used to analyze BW changes, ocular parameters, the number of MCs, degranulation ratio, and protein expression. Pearson's correlation analysis was used to analyze the associations between degranulated MCs and axial elongation, as well as between degranulated MCs and choroidal thinning. The normality of the data distribution was confirmed using the Shapiro–Wilk test (*P* > 0.05). Data analysis was performed using Prism 10 (GraphPad Software, Boston, MA, USA). Statistical significance was set at *P* < 0.05. All experiments, including SD-OCT, histochemistry, immunohistochemistry, flow cytometry, and western blotting, were representative of at least two independent replicates to confirm reproducibility.

## Results

### Increased Choroidal MC Degranulation in LIM Mice

**Figure 1.**
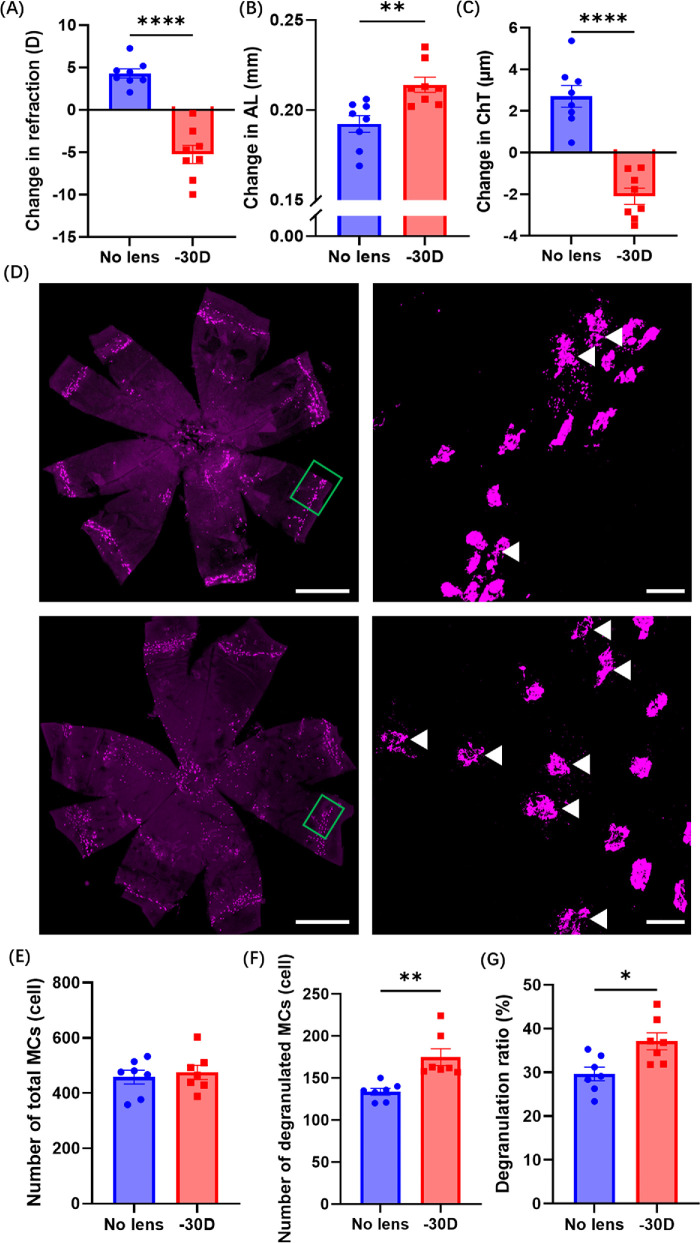
**Increase in degranulated MCs in LIM mice.** (**A**–**C**) Changes in refraction, AL, and ChT in LIM mice (*n* = 8 per group). (**D**) Representative images of choroidal MCs in choroidal flatmounts from LIM mice. The *green rectangles* indicate choroidal MCs. *Scale bars*: 1000 µm. *White arrowheads* indicate degranulated MCs. *Scale bar*: 50 µm. (**E**–**G**) Quantitative analysis of the number of total MCs, number of degranulated MCs, and degranulation ratios in choroidal flatmounts from LIM mice (*n* = 7 per group). Data are shown as mean ± SEM. Statistical analysis was determined using an unpaired two-tailed *t*-test. *P < 0.05, **P < 0.01, ****P < 0.0001.

The LIM mouse model was utilized to investigate changes in choroidal MCs.^25^ Eyes treated with −30 D lenses exhibited a significant myopic shift (*P* < 0.0001), axial length elongation (*P* < 0.01), and choroidal thinning (*P* < 0.0001) compared to control eyes (Figs. 1A–C). As a negative control, no signal was detected in choroidal flatmounts incubated with secondary antibody alone, confirming the specificity of anti-chymase antibody labeling (Supplementary Fig. S1). Choroidal MCs were classified as un-degranulated or degranulated, with the latter characterized by irregular cell shape and visibly released granules (Fig. 1D). Although the number of total MCs did not differ between −30 D lens–treated eyes and control eyes, the number of degranulated MCs and the degranulation ratio were significantly increased in −30 D lens–wearing eyes (*P* < 0.01 and *P* < 0.05, respectively) (Figs. 1E–G). By contrast, the expression levels of chymase and tryptase, the most abundant proteases released by MCs, were comparable between −30 D lens–treated and control choroids (Supplementary Fig. S2). These results suggest that myopic stimuli induced MC degranulation in the choroid without altering overall choroidal MC abundance.

### Inhibition of Myopia Development in Lens-Wearing MC-Deficient Mice

**Figure 2.**
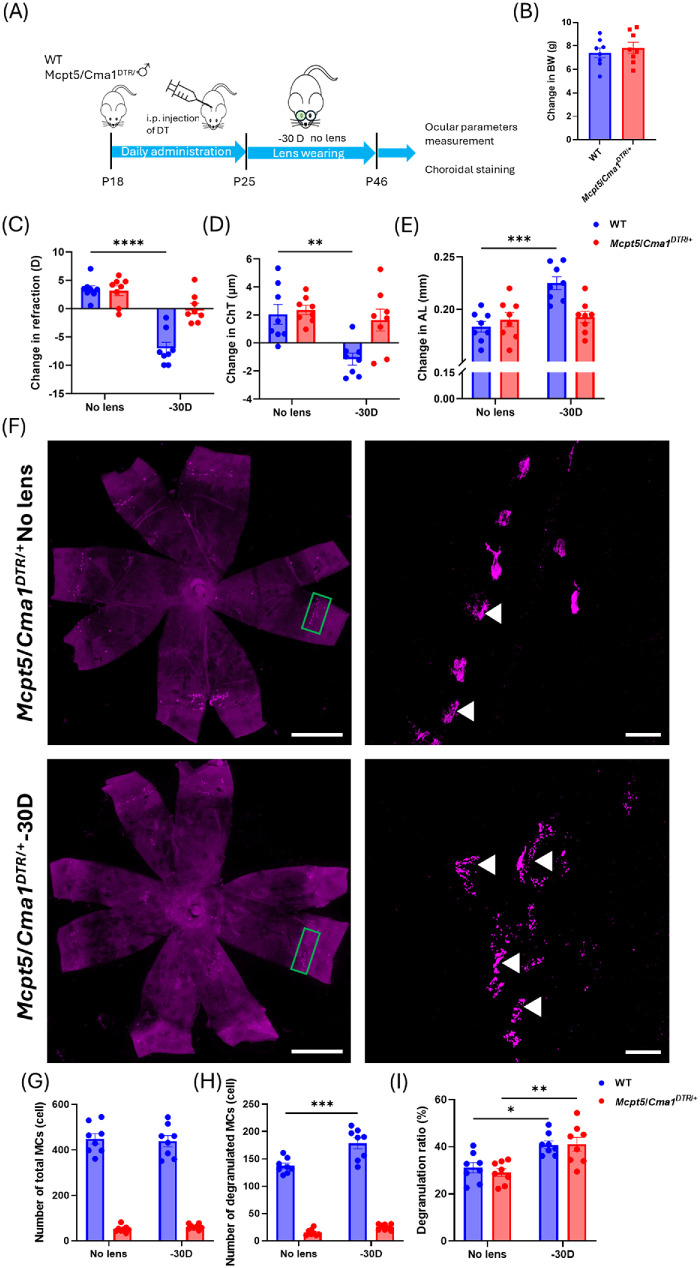
**Inhibition of myopia development in *Mcpt5*/*Cma1^DTR^*^/+^ mice.** (**A**) Timeline of the experimental procedure for *Mcpt5*/*Cma1^DTR^*^/+^ mice and WT mice subjected to LIM. (**B**–**E**) Changes in BW, refraction, AL, and ChT between P25 and P46 following LIM induction (*n* = 8 per group). (**F**) Representative images of choroidal MCs in choroidal flatmounts in *Mcpt5*/*Cma1^DTR/+^* mice subjected to LIM. The *green rectangles* indicate choroidal MCs. *Scale bar*: 1000 µm. *White arrowheads* indicate degranulated MCs. *Scale bar*: 50 µm. (**G**–**I**) Quantitative analysis of the number of total MCs, the number of degranulated MCs, and degranulation ratios in WT mice and *Mcpt5*/*Cma1^DTR/+^* mice following LIM (*n* = 8 per group). Data are presented as mean ± SEM. Statistical analysis was determined using an unpaired two-tailed *t*-test (**B**) and a two-way ANOVA with Tukey's post hoc test (**C**–**E**, **G**–**I**). *P < 0.05, **P < 0.01, ***P < 0.001, ****P < 0.0001.

To investigate the role of MCs in myopia, we generated MC-deficient mice by administering daily intraperitoneal DT injections for 7 days to *Mcpt5*/*Cma1^DTR^*^/+^ mice, which resulted in significant MC depletion in the skin of the ear and abdomen, tongue, and choroid (Supplementary Figs. S3A, S3B), without affecting BW or ocular parameters (Supplementary Figs. S3C–S3G). After DT treatment, *Mcpt5*/*Cma1^DTR^*^/+^ mice and WT littermates underwent LIM (Fig. 2A). BW remained comparable between groups (Fig. 2B). In WT mice, the −30 D lens induced significant myopic changes, including shifts in refraction (*P* < 0.0001), AL (*P* < 0.001), and ChT (*P* < 0.01) (Figs. 2C–E). In contrast, *Mcpt5*/*Cma1^DTR^*^/+^ mice showed no differences between the lens-treated and contralateral eyes and resisted lens-induced myopic changes (Figs. 2C–E).

Immunostaining of choroidal flatmounts revealed that, although the degranulation ratio was significantly increased in lens-wearing eyes compared to the contralateral control in both WT and *Mcpt5*/*Cma1^DTR^*^/+^ mice (*P* < 0.05 and *P* < 0.01, respectively) (Fig. 2I), the number of degranulated MCs was only increased in WT mice (*P* < 0.001) (Fig. 2H). Together, these findings indicate that choroidal MCs are required for myopia development.

### Myopia Was Induced in MC-Deficient Mice With PMC Transplantation

**Figure 3.**
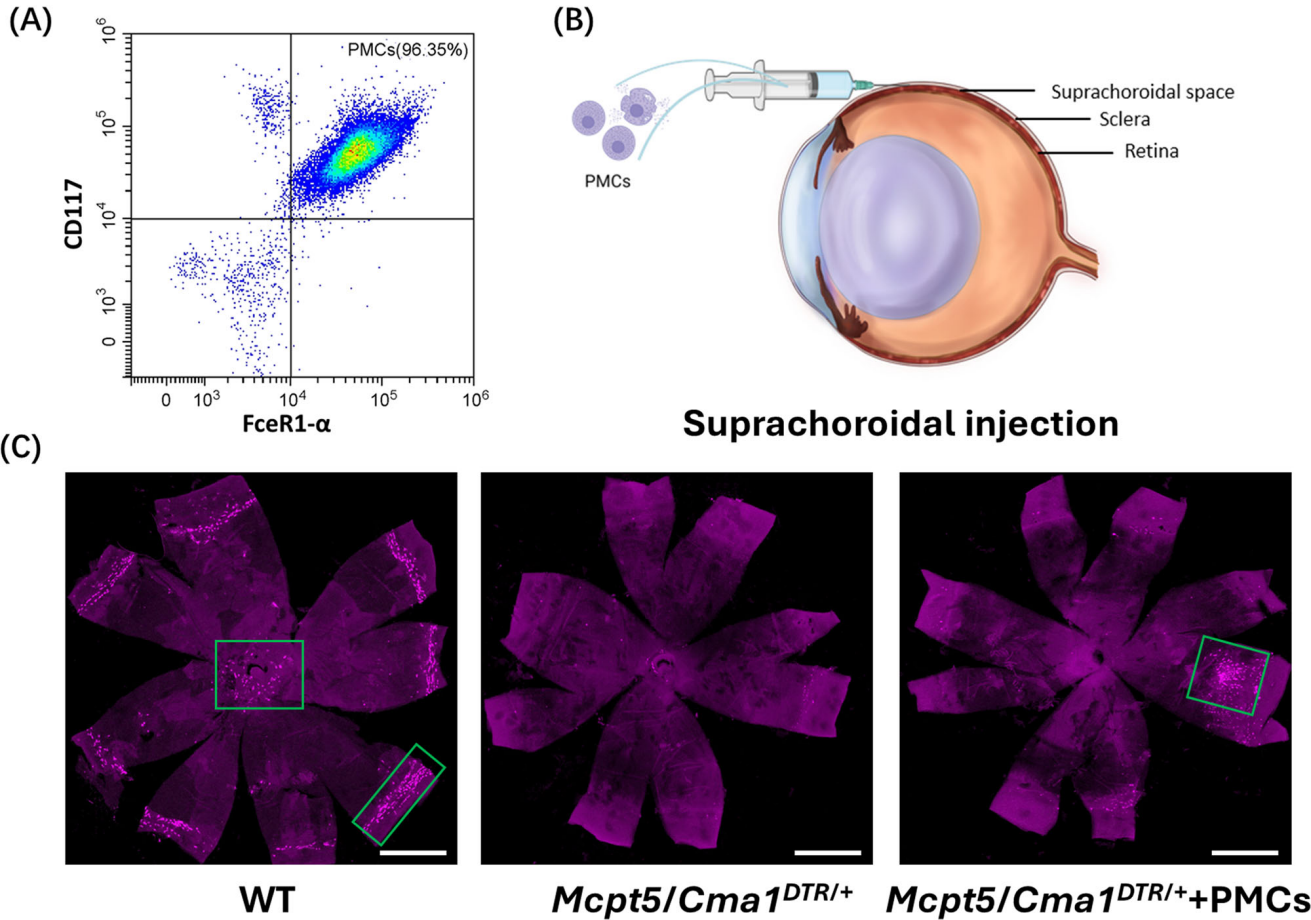
**Suprachoroidal injection of PMCs in *Mcpt5*/*Cma1^DTR^*^/+^ mice.** (**A**) Flow cytometry analysis showing the purity of cultured PMCs, defined as CD117^+^ FceR1α^+^ cells, after 3 weeks of in vitro cultivation. (**B**) Schematic diagram of the suprachoroidal injection of PMCs into *Mcpt5*/*Cma1^DTR^*^/+^ mice. (**C**) Representative images of choroidal MCs in choroidal flatmounts from WT mice treated with PBS, *Mcpt5*/*Cma1^DTR/+^* mice treated with PBS, and *Mcpt5*/*Cma1^DTR/+^* mice treated with PMC. The *green rectangles* highlight choroidal MCs. *Scale bar*: 1000 µm.

To investigate the contribution of choroidal MCs to myopia development, PMCs were injected into the SCS of MC-deficient mice (Fig. 3A). PMCs are mature connective tissue MCs containing abundant histamine and proteases in their granules. The PMCs were cultivated for 3 weeks, achieving a purity of 96% (Fig. 3B). In vitro PMC staining revealed positive chymase expression, indicating their maturation (Supplementary Fig. S4). Seven days post-injection, choroidal flatmounts immunostained with a chymase antibody exhibited a significant increase in chymase signals in PMC-treated *Mcpt5*/*Cma1^DTR^*^/+^ mice compared to PBS-treated *Mcpt5*/*Cma1^DTR^*^/+^ mice (Fig. 3C).

**Figure 4.**
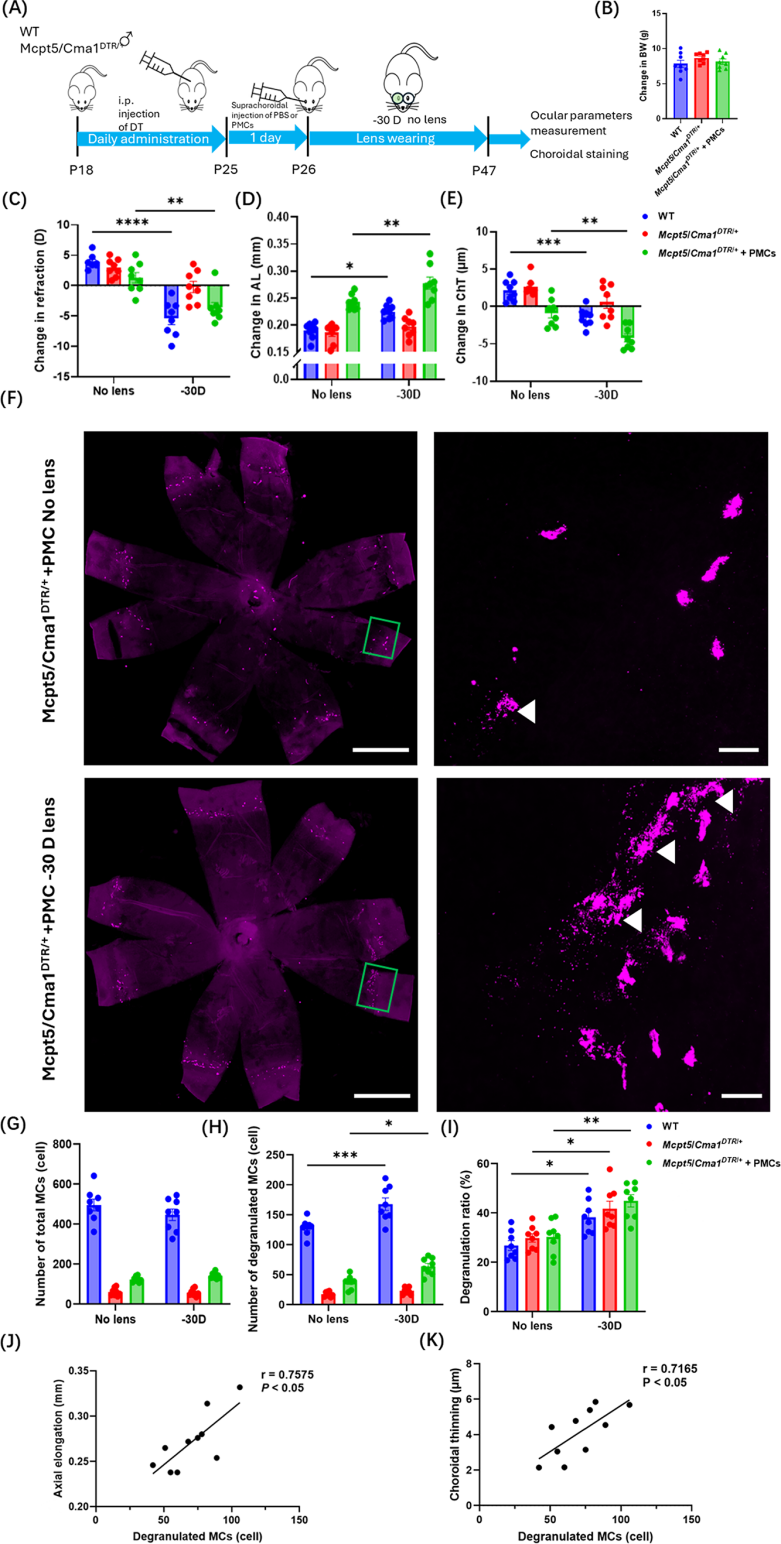
**PMC administration in the choroid contributes to myopia development.** (**A**) Timeline of the experimental procedure for *Mcpt5*/*Cma1^DTR^*^/+^ mice treated with PMCs or PBS and WT mice treated with PBS, all subjected to LIM. (**B**–**E**) Changes in BW (**B**), refraction (**C**), AL (**D**), and ChT (**E**) between P26 and P47 following LIM induction (*n* = 8 per group). (**F**) Representative images of choroidal MCs in choroidal flatmounts from *Mcpt5*/*Cma1^DTR^*^/+^ mice treated with PMCs under LIM conditions. The *green rectangles* highlight choroidal MCs. *Scale bar*: 1000 µm. *White arrowheads* indicate degranulated MCs. *Scale bar*: 50 µm. (**G**–**I**) Quantitative analysis of the number of total MCs (**G**), the number of degranulated MCs (**H**), and degranulation ratios (**I**) in WT mice (PBS-treated) and *Mcpt5/Cma1*^DTR/+^ mice (PBS-treated or PMCs-treated) following LIM (*n* = 8 per group). (**J**, **K**) Correlation between degranulated MC number and axial elongation (**J**) or choroidal thinning (**K**) in −30 D lens–wearing eyes in *Mcpt5*/*Cma1^DTR^*^/+^ mice treated with PMCs (*n* = 10 mice/group). Data are shown as mean ± SEM. Statistical analysis was determined using a one-way ANOVA with Tukey's post hoc test (**B**), a two-way ANOVA with Tukey's post hoc test (**C**–**E**, **G**–**I**), and Pearson's correlation analysis (**J**, **K**). *P < 0.05, **P < 0.01, ***P < 0.001, ****P < 0.0001.

To assess the effect of MC recovery on myopia development, PMC-treated *Mcpt5*/*Cma1^DTR^*^/+^ mice were subjected to LIM, and their ocular parameters and their choroidal MC status were evaluated (Fig. 4A). BW did not differ among the groups (Fig. 4B). In the PMC-treated group, mice exposed to the −30 D lens developed a significant myopic shift in refraction, AL and ChT (*P* < 0.01) (Figs. 4C–E). Immunofluorescence of choroidal flatmounts showed that, despite there being no significant difference in total MC number between −30 D lens–treated eyes and the contralateral eyes in the PMC-treated group, degranulated MC number and MC degranulation (*P* < 0.05 and *P* < 0.01, respectively) were significantly increased in eyes wearing the −30 D lens (Figs. 4F–I). Furthermore, in PMC-treated group, the −30 D lens–wearing eyes showed hallmarks of myopia, including axial elongation (*r* = 0.7575, *P* < 0.05) and choroidal thinning (*r* = 0.7165, *P* < 0.05), positively correlated with the number of degranulated MCs (Figs. 4J, 4K). Together, these results demonstrate that choroidal MCs are a critical component of myopia development.

### MC Degranulation Inhibition Suppressed Myopia Progression by MC Stabilizer Eye Drops

**Figure 5.**
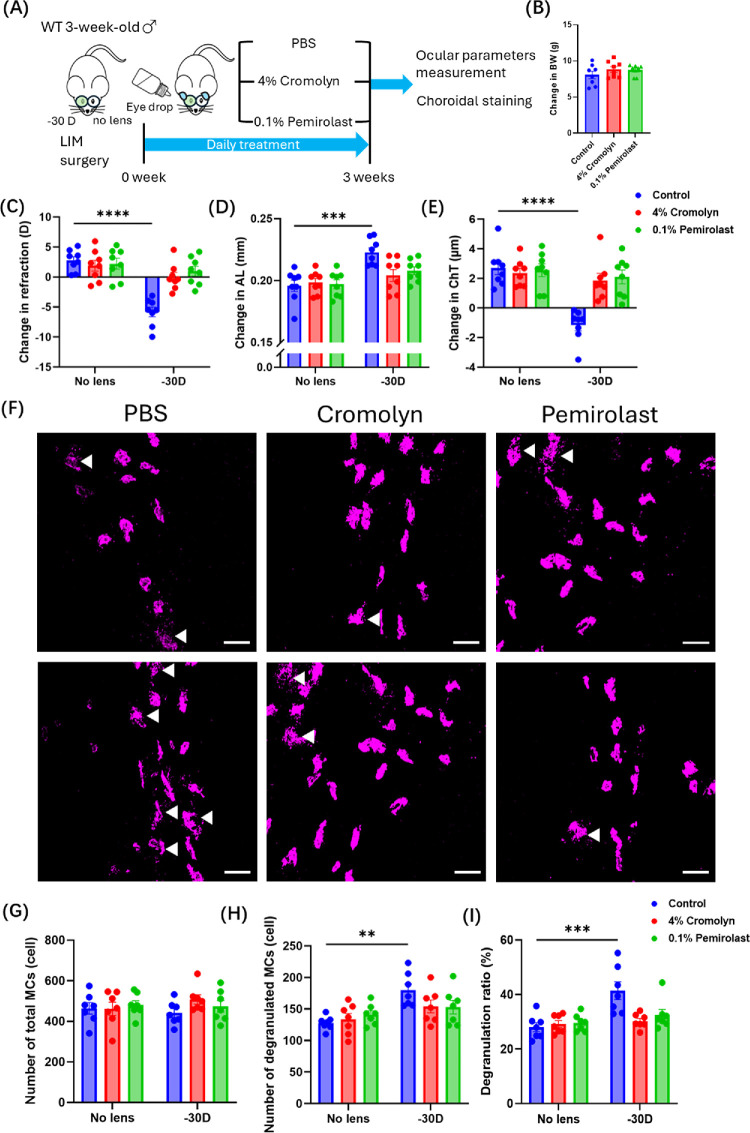
**Daily administration of MC stabilizers suppresses myopia development in LIM mice.** (**A**) Timeline of the experimental procedure for WT mice treated daily with 4% cromolyn, 0.1% pemirolast, or PBS, all subjected to LIM. (**B**–**E**) Changes in BW (**B**), refraction (**C**), AL (**D**), and ChT (**E**) from baseline (0 week) to 3 weeks after LIM induction (*n* = 8 per group). (**F**) Representative images of choroidal MCs in choroidal flatmounts from WT mice treated with 4% cromolyn, 0.1% pemirolast, or PBS under LIM conditions. *White arrowheads* indicate degranulated MCs. *Scale bar*: 50 µm. (**G**–**I**) Quantitative analysis of the number of total MCs (**G**), the number of degranulated MCs (**H**), and MC degranulation ratios (**I**) in WT mice treated with 4% cromolyn, 0.1% pemirolast, or PBS following LIM (*n* = 7 per group). Data are shown as mean ± SEM. Statistical analysis was determined using a one-way ANOVA with Tukey's post hoc test (**B**) and a two-way ANOVA with Tukey's post hoc test (**C**–**E**, **G**–**I**). **P < 0.01, ***P < 0.001, ****P < 0.0001.

Cromolyn and pemirolast eye drops are widely used as MC stabilizers to suppress MC degranulation. The commercial doses for AC are 4% cromolyn and 0.1% pemirolast. In non-LIM mice, administration of cromolyn and pemirolast eye drops for 3 consecutive weeks had minimal effects on CRC, CCT, ACD, VCD, LT, and RT (Supplementary Figs. S5A–S5F). In addition, no significant changes were observed in retinal function (Supplementary Figs. S5G–S5I). These findings indicate that the eye drops had minimal effects on the anterior segment and retina under non-LIM conditions. Cromolyn and pemirolast eye drops were then administered to LIM mice to assess the efficacy in suppressing myopia, and PBS eye drops were administered to the control mice (Fig. 5A). The changes in BW were comparable across all groups (Fig. 5B). In the PBS-treated mice, eyes wearing a −30 D lens developed significant myopic changes (Figs. 5C–E). In contrast, in the cromolyn-treated and pemirolast-treated groups, no significant differences were observed between the lens-wearing and no-lens eyes for the measured ocular parameters (Figs. 5C–E), indicating that MC stabilizer eye drops suppressed LIM development. Subsequently, choroidal MCs were stained after 3 weeks of eye drop treatment. Although the number of degranulated MCs (*P* < 0.01) and the MC degranulation ratio (*P* < 0.001) were increased in eyes treated with the −30 D lens compared with those without lenses in the PBS-treated group, no significant differences were observed between the eyes with and without the −30 D lens in the cromolyn and pemirolast groups (Figs. 5F–I). Consistently, a decrease expression of COL1A1 and an increased expression of MMP-2 were detected in −30 D lens–treated eyes; however, these changes were attenuated by MC stabilizer treatment (Supplementary Figs. S6A–S6D). Collectively, our findings indicate that choroidal MC degranulation caused scleral remodeling and this might be a cause of myopia development.

## Discussion

**Figure 6.**
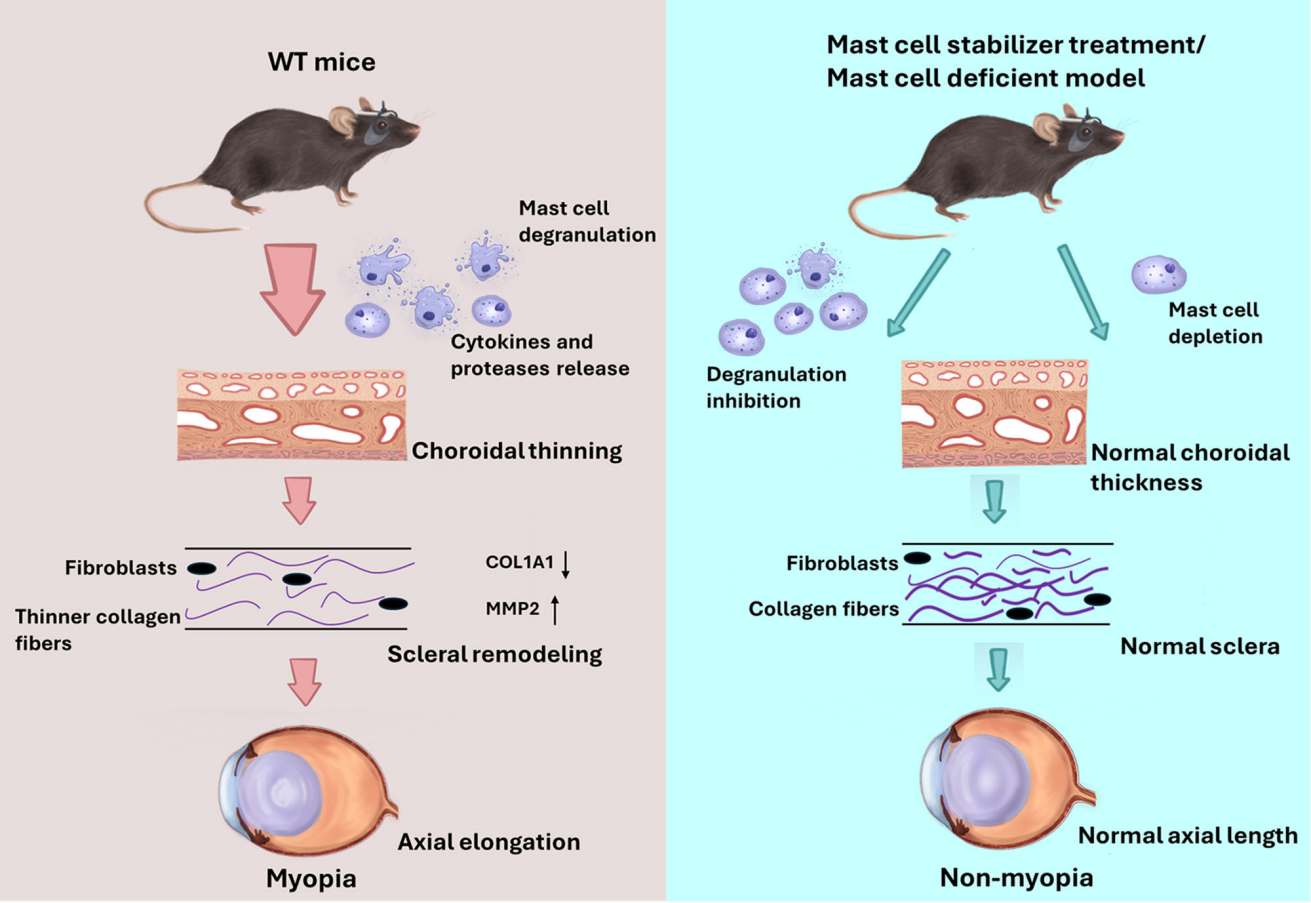
Schematic model illustrating the choroidal MC degranulation in LIM mice and the effects of choroidal MCs and their degranulation on myopia inhibition.

In this study, we employed three distinct murine models, including LIM, MC-deficient, and choroidal MC-reconstituted, to investigate the relationship between choroidal MCs and myopia development. Myopia was not induced when choroidal MCs were absent or when their degranulation was inhibited (Fig. 6). Notably, restoring myopia in MC-deficient mice following choroidal MC reconstitution provided compelling evidence that myopia suppression is specifically attributable to the absence of MCs, confirming the causal role of MCs in myopia progression (Fig. 4).

MCs are widely distributed in the choroid across vertebrate species, whereas in the retina they are notably scarce.^21^ In this study, we observed that the majority of choroidal MCs were located in the limbal region of C57BL/6 mice (Fig. 1D). Furthermore, we demonstrated that pharmacological inhibition of choroidal MC degranulation effectively attenuated myopia progression and suppressed scleral remodeling by preventing the downregulation of COL1A1 and the upregulation of matrix metallopeptidase 2 (MMP-2) (Supplementary Fig. S6). This is consistent with a previous study suggesting that inhibition of MC degranulation attenuates scleral collagen degradation by reducing MMP-2 activity.^31^ Given that MC degranulation has been implicated in fibroblast activation and fibroblast-to-myofibroblast transdifferentiation by increasing MMP-2 activity,^31^^,^^32^ and considering that enhanced myofibroblast differentiation in the sclera is one of the hallmarks of myopia,^33^ our results strongly support the notion that limbal MCs contribute directly to scleral remodeling during myopia development.

MC degranulation initiates inflammatory responses through cytokines such as TNF-α and IL-6.^34^ Elevated levels of these cytokines in the retina and sclera have been observed in myopia models,^35^^,^^36^ where they upregulate MMP-2 expression and promote collagen I degradation, thereby contributing to scleral remodeling and progression of myopia.^36^ These findings indicate that IL-6 and TNF-α released from degranulated MCs may facilitate myopia progression by mediating scleral remodeling. In addition, regions with pronounced MC infiltration and degranulation coincide with choroidal thinning and the loss of the choriocapillaris in GA animal models and patients,^22^^,^^37^ and an in vitro study further demonstrated that chymase induces vascular endothelial cell apoptosis.^38^ Importantly, there is growing clinical evidence that myopia patients show choroidal thinning accompanied by impairment or loss of capillary endothelial components of the choriocapillaris.^39^^,^^40^ Although clinical evidence directly linking choroidal MCs to the development of myopia is currently lacking, these present findings raise the possibility that choroidal MC degranulation might be a cause of myopia development in patients.

MC degranulation has been shown to be induced by both exogenous agents, such as antigens, and endogenous mediators, including neuropeptides.^41^ Although direct evidence linking neuropeptides to myopia development is lacking, they have been implicated in regulating choroidal blood flow and remodeling of ocular tissues.^42^^,^^43^ Accumulating evidence has revealed that there is an increase of pro-inflammatory factors in myopia models and patients, such as upregulation of IL-1β in the sclera and increased IL-6 levels in aqueous humor.^44^^,^^45^ Previous studies have shown that IL-1β is capable of inducing MC degranulation in various tissues including skin and colon.^46^^,^^47^ Notably, IL-1β markedly stimulates the synthesis and release of substance P (SP), a neuropeptide known to directly trigger MC degranulation through Ca^2^⁺ mobilization.^48^^–^^51^ On the other hand, although there is no strong evidence supporting IL-6 as a direct inducer of MC degranulation, prior work indicates that IL-6 promotes an increase of MC reactivity, rendering MCs more prone to degranulate in response to subsequent stimuli.^52^ Taken together, the IL-1β–SP axis may amplify IL-6–activated MC degranulation in the choroid, providing a plausible mechanism by which local inflammatory signals contribute to sustained degranulation. Future studies are warranted to evaluate the expression of cytokines and neuropeptide in myopia models. Nonetheless, our demonstration of MC degranulation in LIM provides a critical foundation for elucidating the underlying mechanisms and for developing targeted interventions for myopia.

In our study, PMCs were injected into the suprachoroidal space to mimic choroidal MCs in myopia development. Our study demonstrated that reconstituted PMCs mainly localized to the peripheral choroid, resembling the distribution of native MCs in C57BL/6 mice. However, the number of reconstituted PMCs was markedly lower, likely due to clearance by the choroidal circulation.^53^ Moreover, although both PMCs and choroidal MCs are classified as connective tissue MCs and exhibit dual positivity for chymase and tryptase,^54^^,^^55^ the amounts of each mediator released upon degranulation are still undetermined. Therefore, although PMC reconstitution offers valuable causal insights, further refinement is needed to more closely replicate the physiological context of native choroidal MCs.

MC stabilizers are pharmacological agents that prevent MC degranulation by cell membrane stabilization.^56^ Cromolyn and pemirolast eye drops, both U.S. Food and Drug Administration (FDA) approved for treating AC, are topical MC stabilizers commonly used in clinical practice.^57^^,^^58^ In our study, topical application of these agents effectively inhibited choroidal MC degranulation and suppressed myopia progression (Fig. 5), without detectable effects on the anterior segment or the retina. According to the prescribing information, topical administration of cromolyn (Akorn, Inc.) and pemirolast (Santen Pharmaceutical Co.) achieves low intraocular and very low plasma concentrations in humans, indicating that topical MC stabilizers are unlikely to substantially disrupt ocular or systemic immune function, although further studies are warranted to confirm their long-term safety. Moreover, accumulating evidence has suggested a strong association between allergic diseases and myopia development.^35^^,^^59^ Clinical studies have consistently reported that children with AC have a higher prevalence of myopia and an increased risk of myopia progression.^60^^,^^61^ These findings emphasize the potential role of allergic responses in the pathogenesis of myopia and the importance of investigating the underlying immunological mechanisms. Taken together, targeting choroidal MC degranulation with MC stabilizers to suppress axial elongation and choroidal thinning may represent a clinically feasible and effective approach to mitigate myopia progression.

In conclusion, we demonstrated that choroidal MCs and their degranulation are crucial in myopia development. Pharmacological inhibition of MC degranulation effectively suppressed myopia progression in a mouse model, indicating a novel and promising therapeutic strategy for myopia control.

## Supplementary Material

Supplement 1
