# Supplementary material for: Choroidal Mast Cells and Their Degranulation Are a Pivotal Trigger for Myopia Development
Source: Invest Ophthalmol Vis Sci. 2025 Nov 12;66(14):22. doi: 10.1167/iovs.66.14.22 (PMC12614256; doi:10.1167/iovs.66.14.22)
Supplement: Supplement 1 [file iovs-66-14-22_s001.docx]

**Supplementary Figures**

**
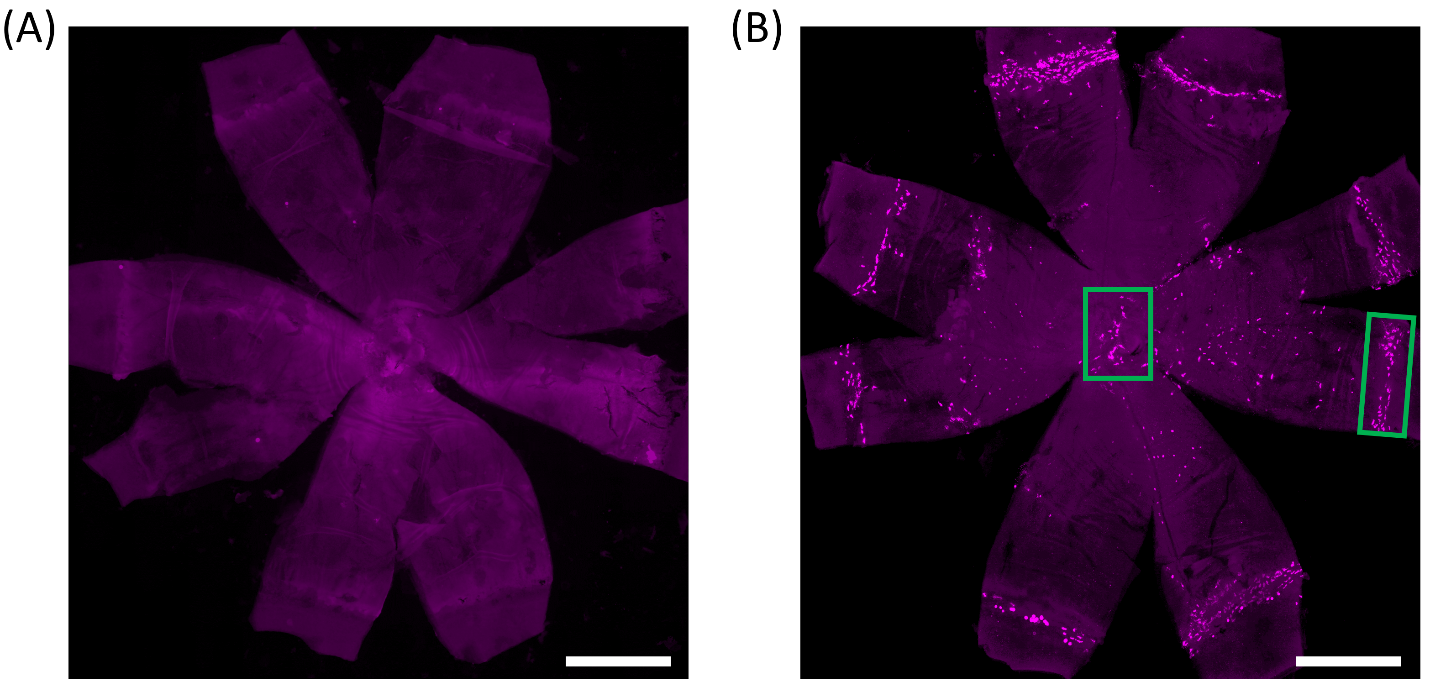
**

**Figure S1. Anti-chymase antibody specifically labels choroidal MCs. (A)** Representative choroidal flat mount image from WT mice processed without anti-chymase antibody (negative control). Scale bar: 1000 μm. **(B)** Representative choroidal flat mount image from WT mice immunostained with the anti-chymase antibody. Green rectangles indicate choroidal MCs. Scale bar: 1000 μm.


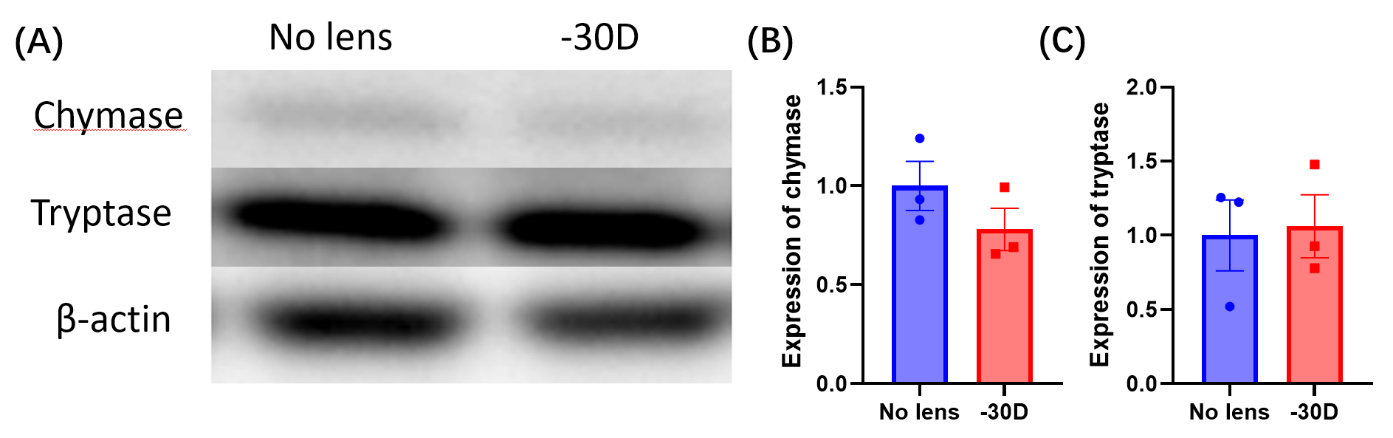


**Figure S2. The expression of chymase and** **tryptase was not changed in LIM mice.** (**A**) Western blot analysis showed the effects of LIM on the expression of chymase and tryptase in choroidal MCs. (**B**) Quantification of the expression of chymase in choroidal MCs between -30 D lens-wearing eyes and control eyes (n = 3 per group, each sample pooled 2 choroids). Statistical analyses were performed using an unpaired two-tailed *t*-test. (**C**) Quantify the expression of tryptase in choroidal MCs between -30 D lens-wearing eyes and control eyes (n = 3 per group, each sample pooled 2 choroids). Data are shown as mean ± SEM. Statistical analyses were performed using an unpaired two-tailed *t*-test.

**
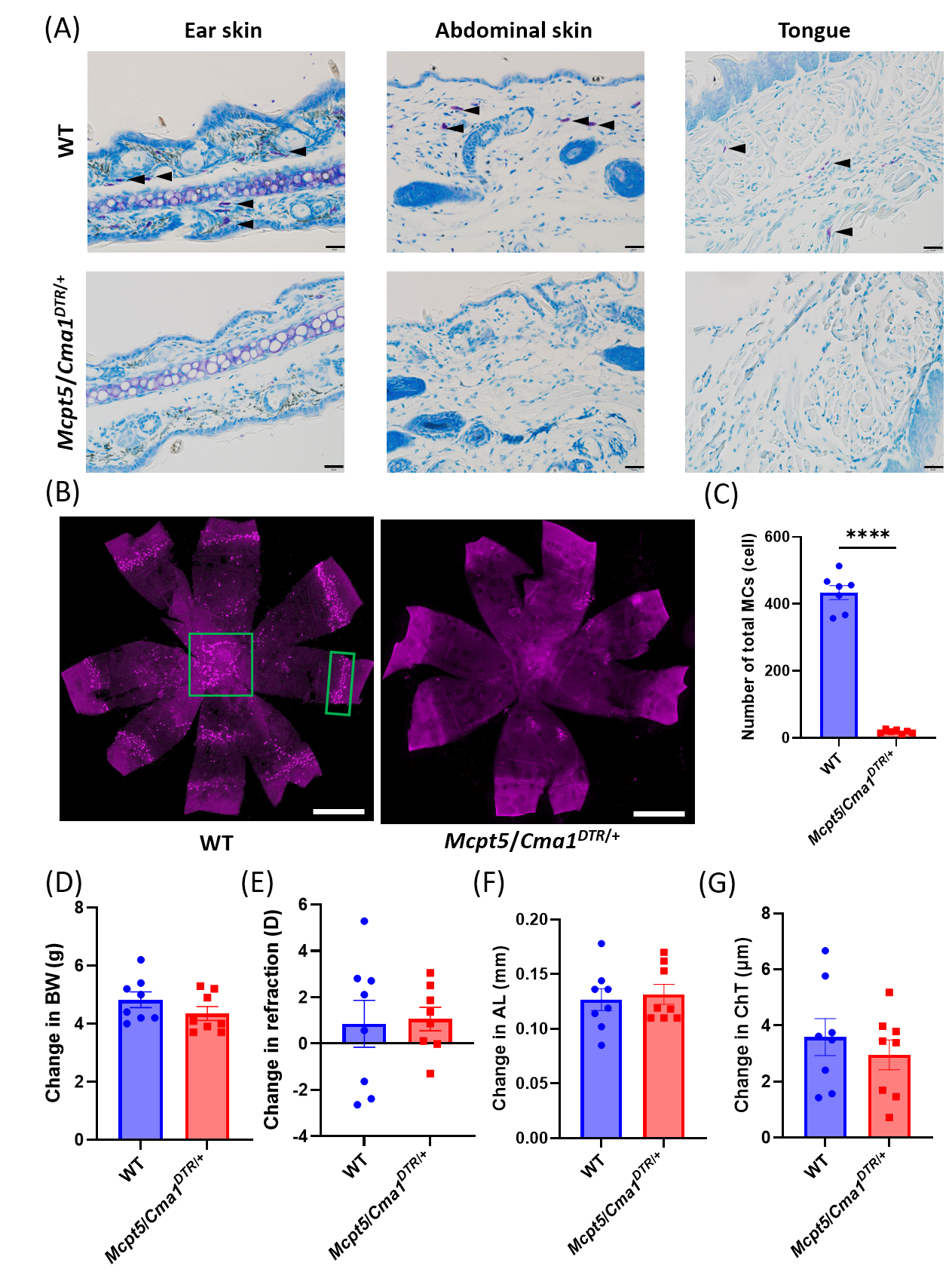
**

**Figure S3. MC depletion has no significant effect on ocular parameter changes in *Mcpt5*/*Cma1^DTR^*^/+^ mice.** (**A**) Representative images of choroidal MCs in the ear skin, abdominal skin, and tongue of WT and *Mcpt5*/*Cma1^DTR^*^/+^ mice after DT treatment. Black arrowheads indicate MCs. Scale bar: 20 μm. (**B**) Representative images of choroidal MCs in choroidal flat mounts of WT and *Mcpt5*/*Cma1^DTR/+^* mice. Green rectangles indicate choroidal MCs. Scale bar: 1000 μm. (**C**) Quantitative analysis of choroidal MCs in WT and *Mcpt5*/*Cma1^DTR^*^/+^ mice (n = 7 per group). (**D-G**) Changes in body weight (BW), refraction, axial length (AL), and choroidal thickness (ChT) between P18 and P25 following DT administration in WT and *Mcpt5*/*Cma1^DTR^*^/+^ mice (n = 8 per group). Data are shown as mean ± SEM. Statistical analyses were performed using an unpaired two-tailed *t*-test. *****P* < 0.0001.

**
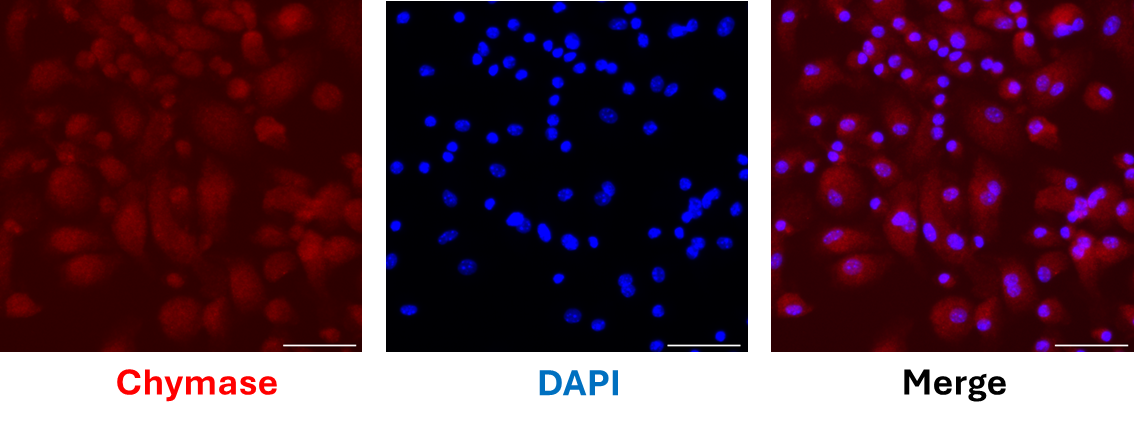
**

**Figure S4. PMCs were chymase-positive cells *in vitro*.** Representative *in vitro* images of PMCs. Scale bar: 50 μm.

**Figure S5**

**
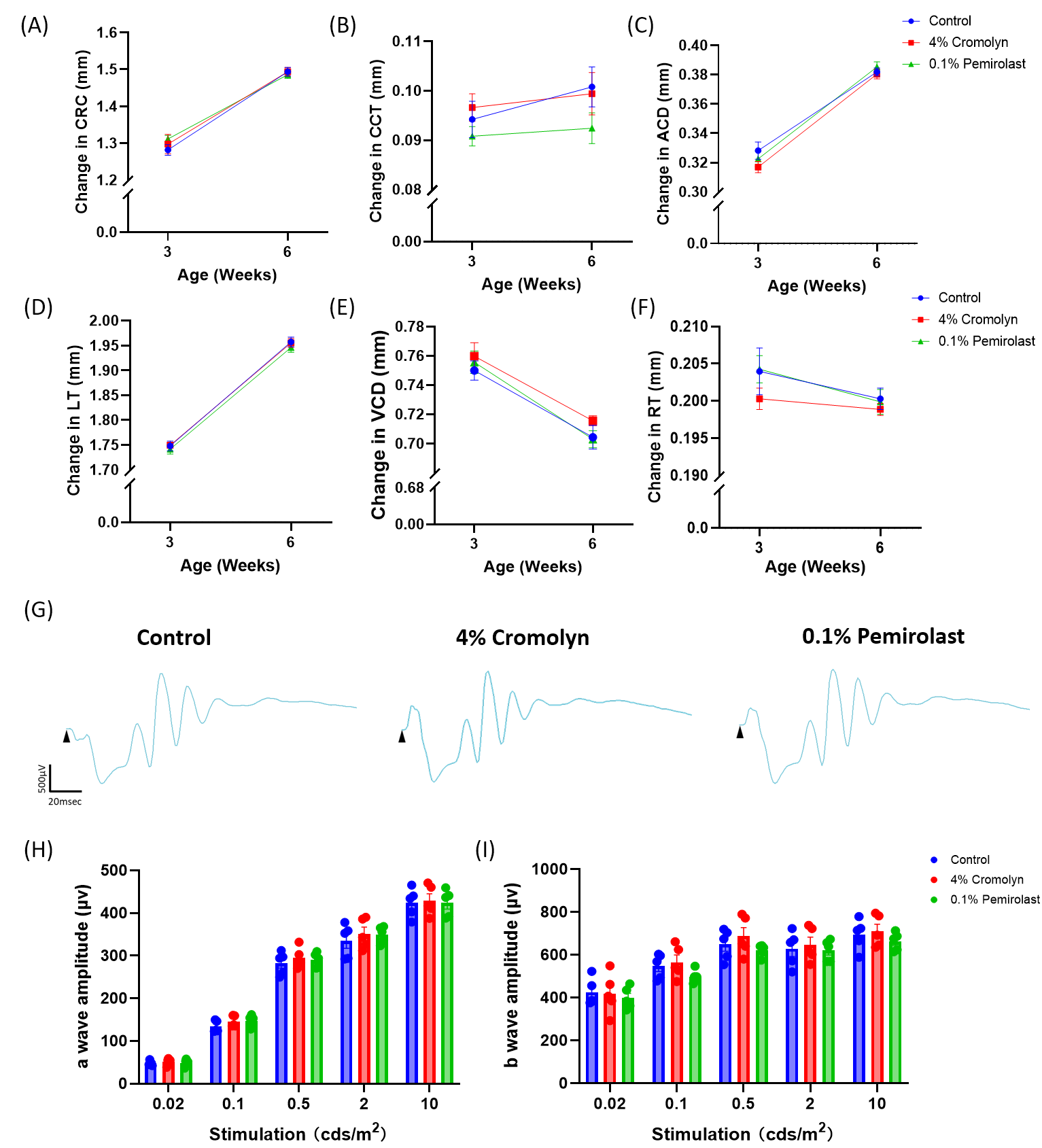
**

**Figure S5. MC stabilizers did not significantly affect anterior segment histology or retinal structure and function in non-LIM mice.** (**A-F**) Changes in corneal radius of curvature (CRC), central corneal thickness (CCT), anterior chamber depth (ACD), lens thickness (LT), vitreous chamber depth (VCD) and retinal thickness (RT) in mice treated with PBS (control), 4% cromolyn or 0.1% pemirolast eye drop. (n = 5 per group). (**G**) Representative ERG waveforms (10 cd ·s/m^2^) of scotopic condition in control, 4% cromolyn-treated or 0.1% pemirolast-treated mice. (**H, I**) Amplitudes of the a-wave and b-wave in control, 4% cromolyn-treated or 0.1% pemirolast-treated mice. (n = 5 per group). Data are presented as mean ± SEM. Statistical analyses were performed using two-way ANOVA with Tukey’s post hoc test.

**
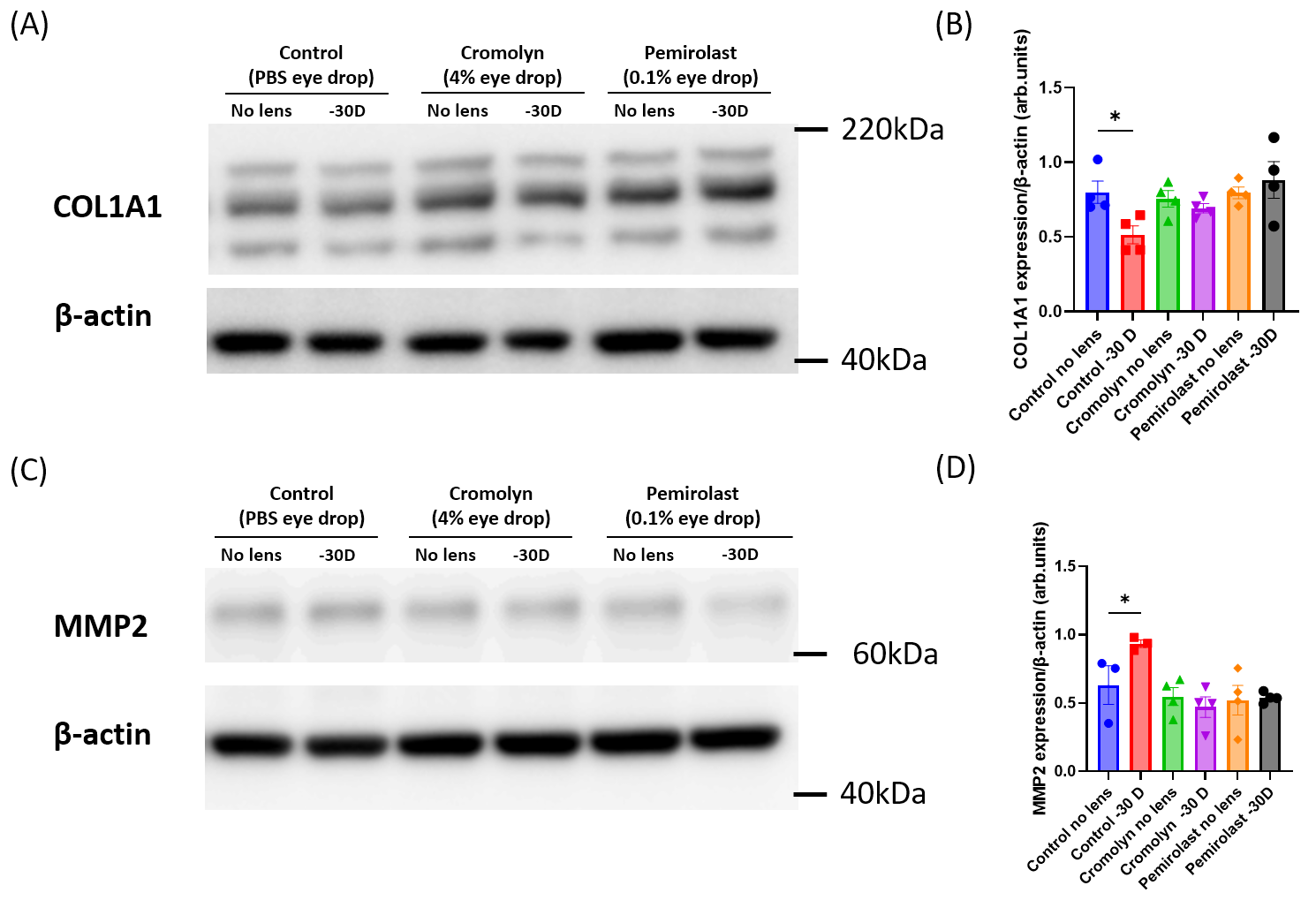
**

**Figure S6. The expression of COL1A1 and MMP2 in sclera in LIM mice with or without MC stabilizer treatment.** (**A**) Western blot analysis showing the effects of MC stabilizer treatment on the expression of COL1A1 in the sclera of LIM mice. (**B**) Quantification of COL1A1 expression in the sclera (n = 4 per group; each sample pooled 2 sclerae). (**C**) Western blot analysis showing the effects of MC stabilizer treatment on MMP2 expression in the sclera of LIM mice. (**D**) Quantification of MMP2 expression in the sclera (control: n = 3; cromolyn: n = 4; pemirolast: n = 4; each sample pooled 2 sclerae). Data are presented as mean ± SEM. Statistical analyses were performed using one-way ANOVA with LSD post hoc test. **P* < 0.05.
